# Porphyromonas Gingivalis Outer Membrane Vesicles Disrupt Trophoblast Mitochondrial FAO and Drive Adverse Pregnancy Outcomes

**DOI:** 10.3390/biomedicines14071640

**Published:** 2026-07-21

**Authors:** Yijia Wang, Siyan Liu, Jiebing Zhang, Ping Ma, Xiaoyuan Li, Yi Liu

**Affiliations:** 1 Laboratory of Tissue Regeneration and Immunology, Department of Periodontics, Beijing Key Laboratory of Tooth Regeneration and Function Reconstruction, School of Stomatology, Capital Medical University, Beijing 100070, China; 2 School of Stomatology, Capital Medical University, Beijing 100070, China; 3 Beijing Institute of Dental Research, School of Stomatology, Capital Medical University, Beijing 100070, China; 4 Department of Stomatology, The Fifth People’s Hospital of Jinan, Jinan 250022, China

**Keywords:** *Porphyromonas gingivalis*, outer membrane vesicles, adverse pregnancy outcomes, mitochondria, fatty acid β-oxidation

## Abstract

**Background:** Periodontitis is increasingly recognised as a significant risk factor for adverse pregnancy outcomes (APOs), yet the mechanisms by which oral pathogens trigger placental dysfunction remain unclear. *Porphyromonas gingivalis* (*Pg*), a keystone periodontal pathogen, secretes outer membrane vesicles (*Pg*-OMVs) that carry virulence factors and can reach distant organs. However, the impact of *Pg*-OMVs on placental trophoblasts and the accompanying metabolic disturbances is poorly understood. This study investigated the effects of *Pg*-OMVs on trophoblast function and metabolism. **Methods:** *Pg*-OMVs were isolated from *Pg* cultures. Pregnant mice were exposed to *Pg*-OMVs in vivo, and placental and uterine weights were recorded. Human trophoblast cells were treated with *Pg*-OMV in vitro. Untargeted metabolomic profiling of placental tissues was conducted using liquid chromatography–mass spectrometry. Fatty acid β-oxidation (FAO) activity, CD36 expression, and mitochondrial integrity were assessed via enzymatic assays, immunoblotting, transmission electron microscopy, mitochondrial membrane potential measurements, and oxygen consumption rate analysis. **Results:** *Pg*-OMVs were internalised by placental trophoblasts, and their presence was associated with significantly reduced placental and uterine weights. Metabolomics revealed a marked accumulation of long-chain acylcarnitines, particularly C20:1 carnitine, indicating impaired FAO. *Pg*-OMV exposure suppressed FAO activity, downregulated the fatty acid translocase CD36, and induced severe mitochondrial dysfunction, evidenced by cristae loss, decreased mitochondrial membrane potential, and diminished oxygen consumption rates. **Conclusions:** *Pg*-OMV compromise placental integrity by disrupting mitochondrial fatty acid β-oxidation, providing a novel mechanistic link between periodontal pathogen-derived vesicles and the pathogenesis of APOs.

## 1. Introduction

Periodontitis is a chronic inflammatory disease caused by an imbalance of the oral microbiota [1]. It affects a large proportion of the global population. Increasing evidence suggests that periodontitis is associated with adverse pregnancy outcomes (APOs), including preterm birth, fetal growth restriction (FGR), and preeclampsia (PE) [2,3]. Previous studies have mainly focused on virulence factors released by periodontal pathogens. After entering the bloodstream, these factors may travel to the placenta and impair placental function [4,5]. However, how these virulence factors target placental tissue and the involved molecular mechanisms remain unclear.

*Porphyromonas gingivalis* (*Pg*) is a major periodontal pathogen. Besides causing local periodontal tissue damage, *Pg* can release virulence factors into the circulation and may therefore contribute to systemic diseases [6]. *Pg* DNA has been identified in the amniotic fluid and placental tissues of women with adverse pregnancy outcomes (APOs) [7,8]. Additionally, virulence factors specific to *Pg*, including lipopolysaccharide (LPS) and gingipain, have been shown to augment the production of cytokines, free radicals, and acute-phase proteins within the uterine cavity, thereby elevating the risk of adverse pregnancy outcomes such as preterm birth [7,9,10,11]. Nevertheless, how Pg drives these pregnancy-related complications at the molecular level remains unclear.

In recent years, an increasing body of evidence indicates that outer membrane vesicles (OMVs) secreted by *Pg* play a significant role in the pathogenesis of various systemic diseases. *Pg*-OMV encompasses various virulence factors, including LPS, phospholipids, and DNA. Due to its distinctive bilayer lipid membrane structure, these vesicles can protect internal virulence factors from degradation and specifically bind to host cells via endocytosis, enabling them to penetrate the blood–brain barrier [12,13]. The pathogenic roles of *Pg*-OMV have been elucidated across various cell types. For example, *Pg*-OMV can infiltrate BV-2 cells and activate the AKT and JNK signalling pathways, thereby inducing neuroinflammation [14]; it can also transport gingival proteases to the liver to reduce insulin sensitivity [15]. Therefore, it is important to investigate how *Pg*-OMV induces APOs.

The placenta is an essential organ that sustains a normal pregnancy, changes in metabolic function could affect placental function [16]. Recent studies indicated that disruptions in fatty acid β-oxidation (FAO) within mitochondria were instrumental in pregnancy-associated complications [17,18]. Among them, trophoblast cells as the fundamental units constituting the placental barrier and executing its functions, it remains unclear whether *Pg*-OMV affects the FAO function of the trophoblast cells. Building upon this foundation, our study investigated the effects of *Pg*-OMV on placental trophoblast FAO function and mitochondrial function. The results provided novel insights into the metabolic origins of pregnancy complications associated with periodontitis and laid the groundwork for further research into the molecular mechanisms underlying adverse pregnancy outcomes related to periodontal disease.

## 2. Materials and Methods

### 2.1. Bacterial Cultures and Pg-OMV Preparation

*Pg* ATCC 33277 was obtained from Shanghai YS Industrial Co., Ltd. (Shanghai, China). The culture procedure and OMV isolation followed previously described methods [19]. Briefly, Pg ATCC 33277 was maintained and propagated in brain–heart infusion broth (Oxoid, Cambridge, UK) supplemented with 0.05% yeast extract, 0.1% vitamin K_1_, and 0.5% hemin under anaerobic conditions (80% N_2_, 10% CO_2_, and 10% H_2_) at 37 °C.

After 2 days of culture, *Pg*-OMVs were isolated from bacterial cultures in the logarithmic growth phase. For freshly cultured *Pg* (about 9 × 10^9^ CFU), the bacterial culture was centrifuged at 10,000× *g* for 15 min at 4 °C to remove the bacteria. The supernatant was filtered through syringe filters (0.22 μm, Millipore, Burlington, MA, USA). The samples underwent ultracentrifugation at 100,000× *g* for 2 h at 4 °C using an Optima L-100 XP Ultracentrifuge (Beckman, Brea, CA, USA) with a type SW32 Ti rotor (Beckman, USA). The OMV fractions were resuspended in 200 μL of phosphate-buffered saline (PBS) and used for subsequent experiments.

Protein concentration was determined using the Bradford assay (Bio-Rad, Hercules, CA, USA), performed in triplicate for each OMV preparation. Then, protein concentrations were measured with the Bradford assay and a SpectraMax i3x (Molecular Devices, San Jose, CA, USA) and transferred to PVDF membranes and incubated with kgp (1:1000, PA5-117637, Invitrogen, Carlsbad, CA, USA) overnight (4 °C). Strips were quantified using ImageJ (1.48v).

### 2.2. OMV Characterization

*Pg*-OMV was characterized through transmission electron microscopy (TEM) and nanoparticle-tracking analysis (NTA). For TEM, *Pg*-OMV suspension (10 μL) was pipetted onto 300-mesh copper grids and adsorption was allowed to proceed for 15 min. After adsorption was complete, 2% polyformaldehyde fixative (10 μL) was pipetted onto the front of the copper mesh and stood for 20 min to fix the sample. Next, 2% phosphotungstic acid solution (10 μL) was pipetted onto the front side of the copper mesh and allowed it to stand for 90 s for staining. After staining, the excess liquid was removed with a strip of filter paper, and then the copper mesh was air-dried at 37 °C in the dark for 30 min. The dried sample was observed using a TEM HT7800 microscope (Hitachi, Tokyo, Japan) and photographs were taken.

For NTA, *Pg*-OMV (15–20 μL) was diluted with PBS to 2 mL, and then the diluted sample was injected into the sample pool using a 1 mL syringe. The sample was then detected using the ZetaView nanoparticle tracking analyzer (Particle Metrix, Ammersee, Germany) with ZetaView software (8.04.02 SP2). Each sample was measured in 3 technical replicates.

### 2.3. Cell Lines and Animals

Healthy C57BL/6 mice (8–10 weeks, 22–25 g) were sourced from Sibeifu Company (Beijing, China). Female mice were selected and paired overnight with males of the same age at a 2:1 ratio. The following morning, vaginal plugs were checked and recorded as embryonic day (E) 0.5. The experimental animals were randomly divided into 2 groups. At E6.5, the *Pg*-OMV group (n = 6) received an intraperitoneal injection of *Pg*-OMV (5 μg/kg), while the control group (n = 6) was given an equivalent volume of PBS [13]. At E14.5, pregnant mice were euthanised by cervical dislocation. Then, the uterus and placenta tissues were carefully extracted for further analysis. The Animal Care and Use Committee at Capital Medical University School of Stomatology (KQYY-202109-006) approved all mouse procedures. All studies followed regulations and ARRIVE guidelines, reducing animal suffering.

The human first-trimester trophoblast cell line (HTR-8/SVneo cells, CL-0765, RRID: CVCL_7162) and HTR-8/SVneo Cell Complete Medium were obtained from Procell (CM-0765, Wuhan, China). The complete medium consisted of RPMI-1640, 10% Fetal Bovine Serum (FBS) and 1% Penicillin–Streptomycin Solution (PS). Cells were cultured at 37 °C in a 5% CO_2_ incubator and subcultured once 80–90% confluence was reached. For *Pg*-OMV exposure experiments, cells were seeded at assay-specific densities and allowed to adhere overnight. The medium was then replaced with fresh complete medium containing *Pg*-OMV at 1 μg/mL [13]. Each experiment included at least three independent biological replicates.

### 2.4. Tracking Pg-OMV In Vivo and In Vitro

*Pg*-OMV (5 μg/kg in vivo; 1 μg/mL in vitro) [13] was labelled with Cy5-E SE (Cy5, 1:300, C5045, UElandy, Suzhou, China) overnight at 4 °C. Unbound dye was removed by Amicon^®^ Ultra Filter (15 mL, Merck, Millipore, Darmstadt, Germany) on second day. In vivo, pregnant mice were injected intraperitoneally with Cy5-labelled *Pg*-OMV. After 6 h, the mice were euthanised by transcardial perfusion with saline. Then, the E14.5 placentas were harvested for frozen sectioning. Subsequently, Cytokeratin 7 (CK7, 1:100, Ab181598, Abcam, Cambridge, UK) was incubated at 4 °C overnight. Following three washes with PBS, AF488-labeled Goat Anti-Rabbit IgG (H+L) (1:500, A0423, Beyotime Technology, Shanghai, China) was applied at 37 °C for 2 h in the dark. The sections were then incubated with DAPI (Beyotime Technology, China) and immediately examined using an automated digital slicing-scanning system.

In vitro, 5 × 10^4^ HTR-8/SVneo cells were seeded on 24-well plates and cultured overnight. On second day, HTR-8/SVneo cells were incubated with Cy5-labeled *Pg*-OMV (1 μg/mL) for 2 h at 37 °C. After two washes with PBS, HTR-8/SVneo cells were treated with Immunol Staining Fix Solution (P0098, Beyotime Technology, China) to preserve cellular structure at room temperature for 20 min. The fixed cells were then washed three times with PBS containing 0.1% Triton X-100 for 5 min per wash. Actin-Tracker Green-488 (C2201; Beyotime Biotechnology, China) was diluted 1:200 in Immunofluorescence Staining Secondary Antibody Dilution Buffer (P0108; Beyotime Biotechnology, China) to prepare the staining solution. Subsequently, 200 μL of the staining solution was added to each well, and the cells were incubated for 30 min at room temperature in the dark. After staining, the cells were washed three times with PBS containing 0.1% Triton X-100 for 5 min per wash. Images were captured using the GE DeltaVision Ultra (GE, Chicago, IL, USA).

### 2.5. Western Blots (Wb)

HTR-8/SVneo cells were treated with *Pg*-OMV (1 μg/mL) for 24 h, as previously described [20]. The cells were then washed twice with ice-cold PBS and lysed in RIPA buffer supplemented with phenylmethylsulfonyl fluoride (PMSF) and a protease inhibitor cocktail. The lysates were incubated on ice for 30 min with intermittent mixing and subsequently centrifuged at 12,000 rpm for 15 min at 4 °C. The clarified supernatant was collected, and protein concentration was determined using the Bradford assay and measured using a SpectraMax i3x microplate reader (Molecular Devices, San Jose, CA, USA).

Equal amounts of protein were separated by electrophoresis and transferred onto PVDF membranes (IPVH00010, Millipore, USA). Next, the following primary antibodies were incubated overnight at 4 °C: anti-α-TUBULIN (1:2000, #3873, Cell Signaling Technology, Danvers, MA, USA) and anti-CD36 (1:1000, YM8274, Immunoway, San Jose, CA, USA). On the second day, after three washes with Tris-Buffered Saline with Tween-20 (TBST, 10 min/per wash), membranes were incubated with HRP goat anti-rabbit/mouse IgG (H+L) (1:5000, SA00001-1/SA00001-2, Proteintech, Rosemont, IL, USA) for 1 h at room temperature. Protein bands were detected using an enhanced chemiluminescence reagent (P10300, NCM Biotech, Suzhou, China) and quantified using ImageJ software version 1.48v. The relative expression of target proteins was calculated after normalization to the appropriate loading control.

### 2.6. Hematoxylin–Eosin (H&E) Staining

Placental tissues collected at E14.5 were fixed overnight in 4% paraformaldehyde at 4 °C, dehydrated, embedded in paraffin, and sectioned at a thickness of 5 μm. The sections were deparaffinized twice in xylene for 5 min each and rehydrated through a graded ethanol series (100%, 100%, 95%, 95%, 80%, and 70%; 3 min at each concentration). After rinsing under running water for 5 min, the sections were stained with hematoxylin for 1 min and counterstained with eosin for 2 min. Subsequently, the sections were dehydrated through graded ethanol solutions (95%, 95%, 100%, and 100%; 30 s at each concentration), cleared in xylene for 10 min, and mounted with neutral balsam. Histopathological alterations in the placental tissues were examined using an automated digital slide-scanning system.

### 2.7. Untargeted Metabolomics

Untargeted metabolomic profiling was performed on placental tissues collected at E14.5 from the control and *Pg*-OMV groups (n = 6 biological samples per group). Immediately after collection, the placental tissues were snap-frozen in liquid nitrogen and stored at −80 °C until analysis. Metabolite extraction and liquid chromatography–mass spectrometry (LC–MS) analysis were conducted by MetWare Biotechnology Co., Ltd. (Wuhan, China). Frozen samples were thawed on ice, and 20 mg of each tissue sample was homogenized at 30 Hz for 20 s. Subsequently, 400 μL of methanol/water (7:3, *v*/*v*) containing an internal standard was added. The mixture was shaken at 1500 rpm for 5 min and incubated on ice for 15 min, followed by centrifugation at 12,000 rpm for 10 min at 4 °C. A 300 μL aliquot of the supernatant was transferred to a new tube and incubated at −20 °C for 30 min. After a second centrifugation at 12,000 rpm for 3 min at 4 °C, 200 μL of the clarified supernatant was transferred to an autosampler vial for LC–MS analysis.

Chromatographic separation was performed using a Waters ACQUITY ultrahigh-performance liquid chromatography system fitted with an ACQUITY Premier HSS T3 column (1.8 μm, 2.1 × 100 mm) maintained at 40 °C. Mobile phase A was water containing 0.1% formic acid, and mobile phase B was acetonitrile containing 0.1% formic acid. The flow rate was 0.4 mL/min, and the injection volume was 4 μL. The gradient program was as follows: 5–20% B from 0 to 2 min, 20–60% B from 2 to 5 min, 60–99% B from 5 to 6 min, held at 99% B from 6 to 7.5 min, returned to 5% B from 7.5 to 7.6 min, and maintained at 5% B until 10 min. The same chromatographic conditions were used for positive- and negative-ion analyses.

Mass spectrometric detection was performed using a Q Exactive high-resolution mass spectrometer operated in positive- and negative-electrospray ionization modes. Data acquisition alternated between full-scan MS and data-dependent MS/MS scans with dynamic exclusion. Full-scan spectra were acquired over an *m*/*z* range of 75–1000 at a resolution of 35,000. The spray voltage was 3.5 kV in positive mode and 3.2 kV in negative mode. The sheath-gas and auxiliary-gas settings were 30 and 5 arbitrary units, respectively; the ion-transfer-tube and vaporizer temperatures were 320 °C and 300 °C, respectively. MS/MS spectra were acquired using stepped collision energies of 30, 40, and 50 V. The signal intensity threshold was 1 × 10^6^ counts per second, the 10 most intense precursor ions were selected for fragmentation, and the dynamic-exclusion duration was 3 s.

Raw data were processed for peak detection, retention-time alignment, peak integration, normalization, and metabolite annotation using the MetWare in-house database and public metabolite databases. Unsupervised principal component analysis (PCA) was performed using the prcomp function in R (version 4.1.2) after unit variance scaling. Hierarchical cluster analysis (HCA) of samples and metabolites and Pearson correlation coefficients between samples were calculated in R and visualized using the ComplexHeatmap package (version 2.9.4); normalized metabolite signal intensities were unit variance-scaled before HCA heatmap visualization. For two-group comparisons, differential metabolites were defined by a variable importance in projection (VIP) score > 1 and *p* < 0.05 (Student’s *t*-test). VIP scores were obtained from orthogonal partial least-squares discriminant analysis models generated using the MetaboAnalystR package after log_2_ transformation and mean centering.

### 2.8. FAO Activity Test

The FAO activity was tested by Fatty Acid Oxidation (FAO) Colorimetric Assay Kit (E-BC-K784-M, Elabscience Biotechnology Co., Ltd., Wuhan, China). Collected in vivo and in vitro samples were homogenized or lysed in the extraction buffer provided with the kit. After centrifugation (10,000× *g*, 15 min, 4 °C), the supernatant was collected for FAO activity measurement.

FAO activity was determined by measuring the absorbance of an NADH-coupled chromogenic product generated during the β-oxidation reaction. For each reaction, 50 μL of sample, 165 μL of substrate, 145 μL of reaction working solution, and 20 μL of chromogenic reagent were sequentially added to the wells. The reaction mixture was thoroughly mixed and incubated at 37 °C for 30 min. Absorbance was subsequently measured at 450 nm using a microplate reader. Each sample was tested in technical triplicate.

### 2.9. Mitochondrial Ultrastructure of HTR-8/Svneo Cells

After *Pg*-OMV treatment, HTR-8/SVneo cells were collected and fixed with 2.5% glutaraldehyde at 4 °C for 2 h. Samples were then post-fixed with 1% osmium tetroxide for 2 h, dehydrated through graded ethanol, and embedded in epoxy resin. Ultrathin sections of 50–70 nm were prepared using an ultramicrotome and double-stained with uranyl acetate and lead citrate (10 min). Mitochondrial ultrastructure was observed using a JEM-1400Flash transmission electron microscope (Toyoake, Japan).

### 2.10. Mitochondrial Membrane Potential Analysis

Mitochondrial membrane potential (ΔΨm) was evaluated using a JC-1 fluorescence probe kit (E-CK-A301, Elabscience, China). After treatment with *Pg*-OMV, HTR-8/SVneo cells were collected, counted, and 5 × 10^5^ cells were taken. The cells were then centrifuged at 300× *g* for 5 min, and the supernatant was discarded. The cell pellet was resuspended in 500 μL of JC-1 working solution and incubated at 37 °C for 20 min in the dark. After incubation, the cells were centrifuged again at 300× *g* for 5 min, the supernatant was removed, and the cells were washed once with 1 × JC-1 Assay Buffer (centrifugation at 300× *g*, 5 min) to remove excess dye. Finally, the cells were resuspended in an appropriate volume of 1 × JC-1 Assay Buffer for flow cytometry analysis. JC-1 fluorescence was detected using an LSR Fortessa flow cytometer (BD Biosciences, Milpitas, CA, USA). JC-1 aggregates, which emit red fluorescence, indicate high mitochondrial membrane potential (ΔΨm), whereas JC-1 monomers, which emit green fluorescence, indicate decreased ΔΨm. Thus, the red/green fluorescence ratio was used to estimate ΔΨm. Flow cytometry data were analyzed using FlowJo (v10.8).

### 2.11. Seahorse Assay

Mitochondrial respiration was assessed by measuring the oxygen consumption rate (OCR) using the Seahorse XF Cell Mito Stress Test Kit (103015-100, Seahorse Bioscience, Chicopee, MA, USA) and a Seahorse XFe24 Extracellular Flux Analyzer (102340-001, Seahorse Bioscience, USA). HTR-8/SVneo cells (2 × 10^4^ cells/well) were cultured into Seahorse XF24 cell culture microplates and allowed to attach overnight.

Before the assay, the culture medium was replaced with Seahorse XF assay medium adjusted to pH 7.4. Cells were then equilibrated in a non-CO_2_ incubator at 37 °C for 1 h. During OCR measurement, oligomycin, FCCP, and rotenone/antimycin A were sequentially injected according to the assay protocol. Basal respiration, ATP-linked respiration, maximal respiration, and non-mitochondrial respiration were calculated using Seahorse Wave software (version 10.1.0).

### 2.12. Statistical Analysis

Statistical analyses were conducted with GraphPad Prism 9 (GraphPad Software, San Diego, CA, USA). Results were shown as means ± standard deviation. Data were normally distributed and had equal variances, as confirmed by the Shapiro–Wilk test. For comparisons between two groups, unpaired two-tailed *t*-tests were used. A *p*-value < 0.05 was deemed statistically significant. All measurements were performed in triplicate.

## 3. Results

### 3.1. Characterization of Pg-OMV

**Figure 1:**
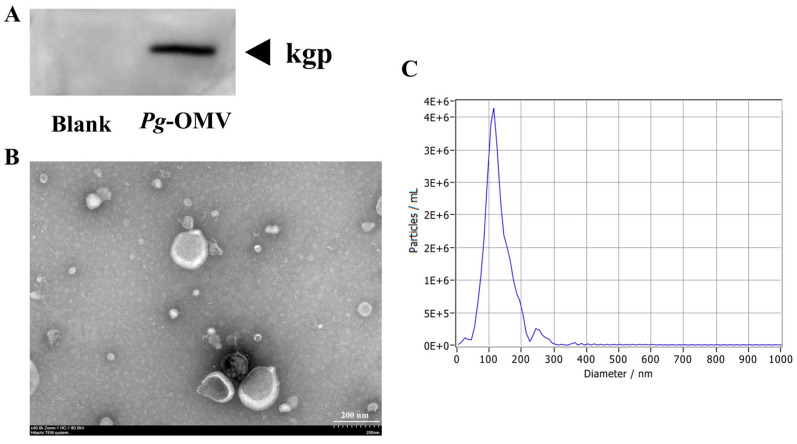
The acquisition and characterization of *Pg*-OMV. (**A**) The protein level of kgp was assessed with WB; (**B**) *Pg*-OMV morphology was observed using TEM; (**C**) *Pg*-OMV size distribution was detected by NTA.

We first characterized the morphology, size, and protein composition of *Pg*-OMV. TEM revealed that *Pg*-OMV exhibited a characteristic spherical bilayer structure, which was in line with the morphological characteristics of OMV (Figure 1B). NTA showed that the mean particle diameter was 115.7 ± 4.1 nm (Figure 1C), which was within the reported size range of OMVs from Gram-negative bacteria, usually about 20–300 nm [21]. WB was conducted to confirm the presence of *Pg*-specific cargo. As shown in Figure 1A, lysine-specific gingipain (Kgp) was significantly more abundant in the *Pg*-OMV group compared to the blank control. These findings demonstrated that *Pg*-OMV was successfully isolated and exhibited the expected morphology, size distribution, and bacterial protein cargo.

### 3.2. Pg-OMV Induced APOs and Invaded Placental Trophoblasts

**Figure 2:**
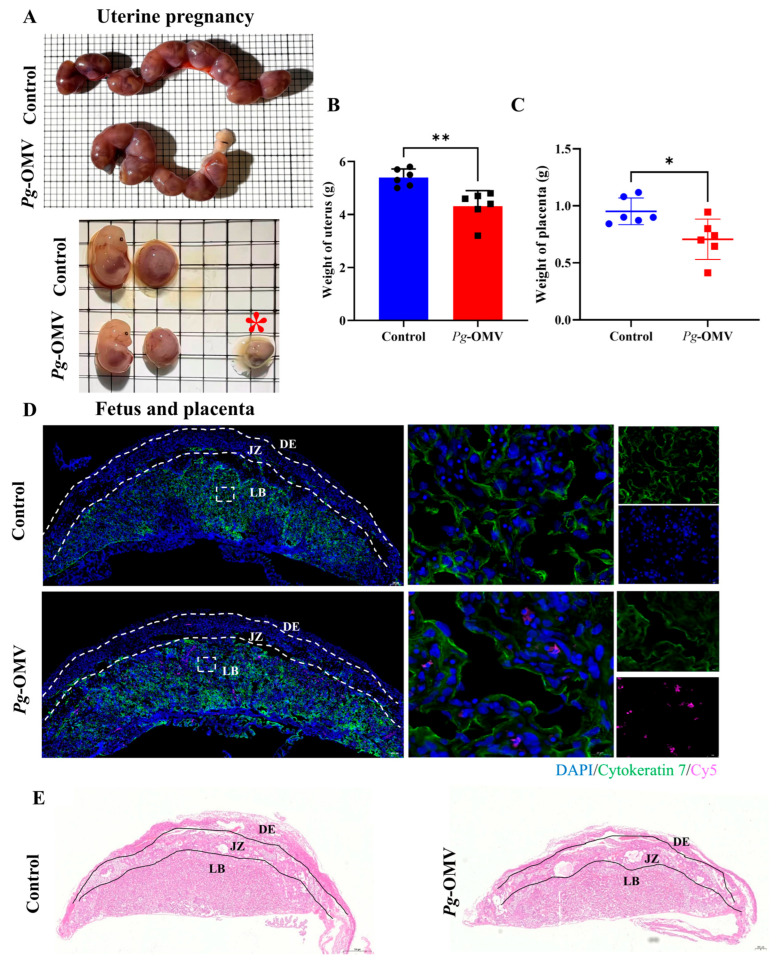
*Pg*-OMV invaded the placenta. (**A**) The diagram of the uterus and placenta at E14.5 (Red *, stillborn fetus.); (**B**,**C**) The weight of the uterus and placenta at E14.5 (n = 6); (**D**) IF showing that *Pg*-OMV was localized in the placental labyrinth layer compared with control group (Cy5: red fluorescent signals; Cytokeratin 7: green fluorescent signals; DAPI, blue signals; left: scale bar 200 μm; right: scale bar 20 μm); (**E**) Representative H&E staining of placental tissues from the control and *Pg*-OMV mice (scale bar 200 μm). DE: Decidua layer; JZ: Junction zone; LB: Labyrinth layer; * *p* < 0.05, ** *p* < 0.01, compared with the negative control group.

To assess whether *Pg*-OMVs affected placental development in vivo, pregnant mice were treated with *Pg*-OMVs, and samples were collected at E14.5. As shown in Figure 2A–C, both uterine weight and placental weight were significantly lower in the *Pg*-OMV group than in the control group. One stillborn fetus was also observed after *Pg*-OMV exposure, suggesting impaired gestational tissue growth and reduced fetal viability.

Because trophoblasts are the main cell type in the labyrinth layer and are important for maintaining placental structure and function [22], we next examined the localization of *Pg*-OMVs in placental tissues. Immunofluorescence staining showed that Cy5-labeled *Pg*-OMVs strongly colocalized with CK7-positive trophoblasts in the placental labyrinth layer (Figure 2D). This result indicated that *Pg*-OMVs could reach and associate with placental trophoblasts in vivo. H&E staining further showed that the thickness of the labyrinth layer was markedly reduced in the *Pg*-OMV group (Figure 2E). Together, these findings suggested that *Pg*-OMV might compromise placental architecture by disrupting trophoblast organization and viability.

### 3.3. Pg-OMV Exposure Disrupted Fatty Acid Metabolism in Placentas

**Figure 3:**
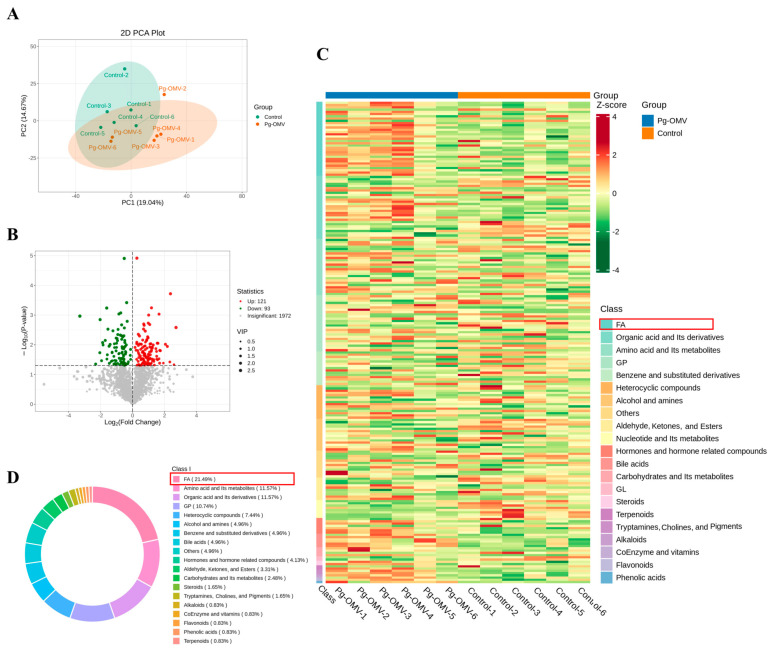
Metabolomic alterations induced by *Pg*-OMV exposure. (**A**) PCA Plot; (**B**) Differential Metabolite Volcano Plot; (**C**) Heatmap of Differential Metabolite Clusters; (**D**) The circular diagram of metabolite categories elevated in the *Pg*-OMV group.

**Figure 4:**
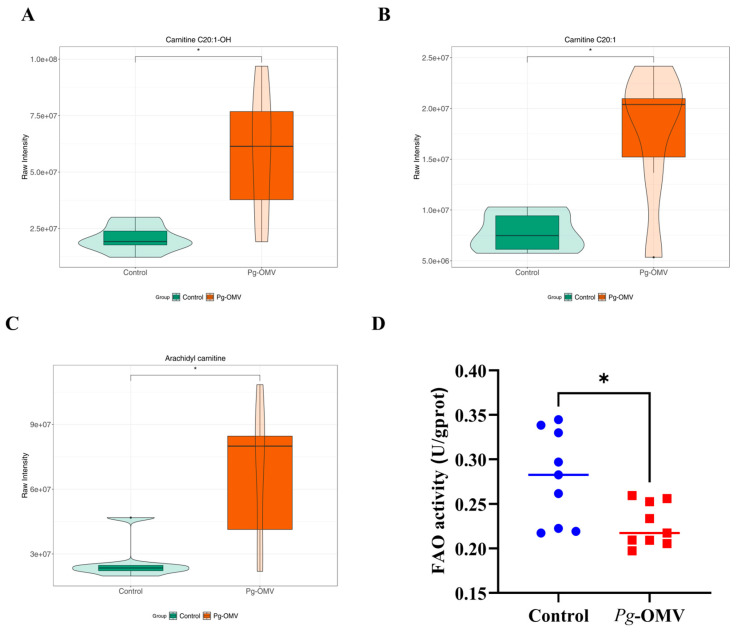
LCAC accumulation and reduced FAO activity in *Pg*-OMV-exposed placental tissues. (**A**–**C**) Violin plot of differential LCACs metabolites. (**D**) ELISA assay for FAO activity on E14.5 placenta tissues. * *p* < 0.05 compared with the negative control group.

As metabolic homeostasis is essential for normal placental development [23], we performed an untargeted metabolomic analysis of placental tissues from control and *Pg*-OMV-exposed placenta. Principal component analysis (PCA) showed a moderate separation in global metabolic profiles between *Pg*-OMV group and control group, suggesting *Pg*-OMV induced changes in the placental metabolome (Figure 3A). Further quantitative analysis identified a total of 121 significantly upregulated metabolites in *Pg*-OMV-treated placentas (Figure 3B). To facilitate the observation of variation patterns in relative metabolite abundances, we applied unit Variance Scaling (UV) scaling to the raw relative abundances of the differential metabolites. The results showed that fatty acids (FAs) were significantly elevated in the placentas treated with *Pg*-OMV and accounted for 21.19% of the differential metabolites (Figure 3C,D). Further profiling the FA-related metabolites revealed a marked accumulation of multiple long-chain acylcarnitines (LCACs), particularly C20:1 carnitine, C20:1-OH carnitine, and arachidyl carnitine, in the placentas exposed to *Pg*-OMV (Figure 4A–C). Since the increase in acylcarnitines was closely related to the inhibition of FAO function [24], we evaluated the activity of FAO by ELISA. The result demonstrated that *Pg*-OMV significantly inhibited FAO activity in placental tissues (Figure 4D). These results suggested that disrupted FAO might represent a critical mechanism underlying *Pg*-OMV-induced placental dysfunction.

### 3.4. Pg-OMV Suppressed FAO Function in HTR-8/SVneo Cells

**Figure 5:**
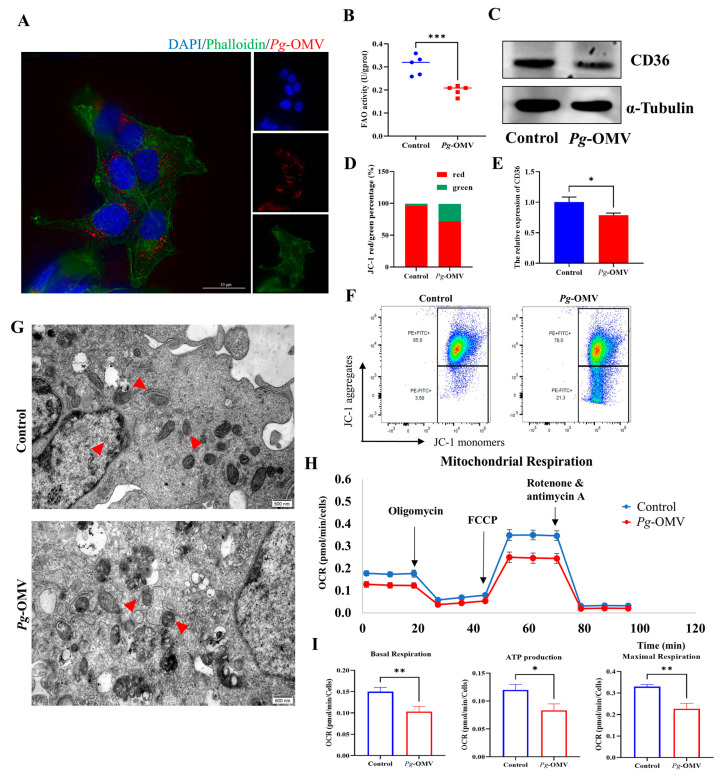
*Pg*-OMV suppressed FAO and was associated with mitochondrial dysfunction in HTR-8/SVneo cells. (**A**) IF showed *Pg*-OMV was internalized by HTR-8/SVneo cells (Cy5: red fluorescent signals; Phalloidin: green fluorescent signals; DAPI: blue signals; scale bar 10 μm). (**B**) ELISA assay for FAO activity; (**C**,**E**) The protein levels of CD36 were determined by WB and quantitative analysis; (**D**,**F**) JC-1 flow cytometric analysis and statistical data of HTR-8/SVneo cells; (**G**) Mitochondrial morphology observed by TEM (magnification: 25,000×, scale bar 500 nm); (**H**,**I**) OCR were measured by Seahorse Bioscience XF24 analyzer in HTR-8/SVneo cells. * *p* < 0.05, ** *p* < 0.01, *** *p* < 0.001 compared with the negative control group.

To further validate the effect of *Pg*-OMV on FAO in HTR-8/SVneo cells. First, we examined the internalization of *Pg*-OMV into trophoblast cells. As shown in Figure 5A, Cy5-labeled *Pg*-OMV were readily detected within the cytoplasm of HTR-8/SVneo cells following 2 h of co-culture, confirming that *Pg*-OMV were efficiently internalized by trophoblasts in vitro. Figure 5B showed that *Pg*-OMV exposure significantly inhibited FAO activity. As a receptor with diverse biological functions, CD36 facilitates the recognition and internalization of fatty acids with long carbon chains and contributes to the regulation of FAO [25]. In our results, WB revealed that *Pg*-OMV treatment reduced CD36 protein expression in HTR-8/SVneo cells (Figure 5C,E), further indicating that *Pg*-OMV inhibited FAO activity.

### 3.5. Pg-OMV Induced Mitochondrial Dysfunction in HTR-8/SVneo Cells

**Figure 6:**
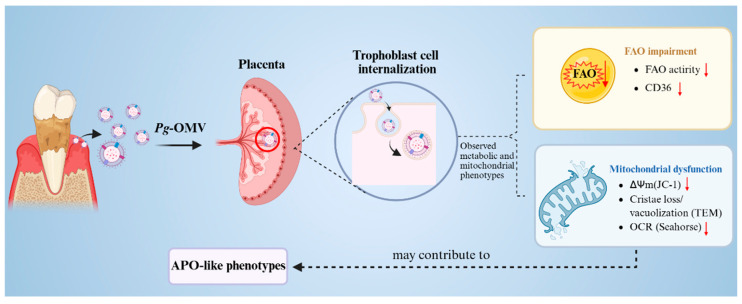
Schematic summary of *Pg*-OMV induced trophoblast injury. *Pg*-OMV reached the placenta and was internalized by trophoblasts, resulting in impaired FAO and mitochondrial dysfunction (The red downward arrows indicated: reduced FAO activity, CD36 expression, ΔΨm, and OCR associated with *Pg*-OMV exposure).

Since FAO primarily takes place in mitochondria, we further evaluated mitochondrial functions in HTR-8/SVneo cells. As shown in Figure 5D,F, flow cytometric analysis using the JC-1 fluorescent probe revealed a decrease in mitochondrial membrane potential following *Pg*-OMV exposure. Consistently, TEM observation revealed obvious mitochondrial damage, including reduced or disrupted cristae and vacuole-like degeneration (Figure 5G). To further evaluate the functional consequences of these mitochondrial alterations, we measured the oxygen consumption rate (OCR) using a Seahorse XF analyzer. *Pg*-OMV exposure led to a marked reduction in OCR, as reflected by decreased basal respiration, maximal respiration, and ATP production in HTR-8/SVneo cells (Figure 5H,I). These data supported that *Pg*-OMV exposure was associated with mitochondrial dysfunction in HTR-8/SVneo cells. Collectively, our results showed that *Pg*-OMV were internalized by placental trophoblasts and were associated with impaired FAO and mitochondrial dysfunction. The latter was supported by reduced mitochondrial membrane potential, induced mitochondrial ultrastructural damage, and decreased OCR. These findings may contribute to the placental dysfunction and APO-like phenotypes observed following *Pg*-OMV exposure (Figure 6).

## 4. Discussion

In this current work, we found that *Pg*-OMV was sufficient to impair placental development and induce trophoblast injury. Specifically, *Pg*-OMV exposure reduced placental number and weight in pregnant mice. Importantly, placental metabolomic profiling revealed marked disturbances in fatty acid metabolism following *Pg*-OMV exposure, particularly the accumulation of carnitines. Consistent with this metabolic alteration, cellular experiments showed that *Pg*-OMV downregulated CD36 expression, and induced mitochondrial damage. Collectively, these findings supported a model in which *Pg*-OMV compromises placental development by suppressed FAO and mitochondrial integrity.

As the cellular hub of energy metabolism, mitochondria are intimately associated with fatty acid utilization. In the placenta, effective fatty acid management is essential not only for maintaining energy balance but also for the survival and function of trophoblasts [26,27]. Therefore, blocking FAO was likely to cause significant metabolic stress in these cells. Previous studies have shown only that *Pg*-OMVs could be internalised by HTR-8/SVneo cells [20], their impacts on trophoblast biological functions, particularly on fatty acid metabolism and mitochondrial integrity, remain largely unknown.

In the current study, untargeted metabolomic analysis demonstrated that fatty acid metabolism was significantly disrupted in placentas exposed to *Pg*-OMV, with carnitine C20 among the most notably elevated metabolites. Acylcarnitines are amino acid-derived compounds that mediate the transport of acyl groups across cellular membranes for β-oxidation and ATP production, thereby exerting direct or indirect regulation over numerous physiological processes [28]. Recent studies have shown that excessive acylcarnitine accumulation, resulting from impaired FAO, could cause mitochondrial damage, disrupt respiratory function and inhibit FAO activity [29]. Consistent with this, existing research has shown that reduced placental FAO leads to lipid buildup, which causes lipotoxicity and metabolic issues that negatively impact the fetus during the perinatal and postnatal periods [30]. Similar mechanisms have been observed in other pathological contexts: ischemia reduces FAO, leading to the accumulation of long-chain acylcarnitines in cardiomyocyte mitochondria [31]; and GPX2 elevates acylcarnitine levels by interfering with lipid droplet formation and lipid homeostasis, thereby compromising mitochondrial function in gastric cancer cells [32].

Given these lines of evidence, we further investigated whether *Pg*-OMV directly affected mitochondrial function in trophoblast cells. Notably, *Pg*-OMVs have been reported to exacerbate diabetic retinopathy by inducing mitochondrial damage and accelerating endothelial dysfunction in HRMECs [33]. In our study, *Pg*-OMV caused obvious mitochondrial ultrastructural damage, significantly reduced mitochondrial membrane potential and oxidative phosphorylation (OXPHOS) in trophoblast cells, indicating substantial impairment of mitochondrial integrity and function. Collectively, these findings suggested that *Pg*-OMV inhibits FAO in trophoblast cells and compromises mitochondrial function, which may help explain the APOs associated with *Pg*-OMV exposure.

Another important finding of the present study was the reduction in CD36 expression following *Pg*-OMV treatment. CD36 is a multifunctional fatty acid translocase involved in lipid uptake and intracellular metabolic adaptation, and its dysregulation has been linked to disturbed lipid handling in several pathological conditions [34]. Intrauterine growth restriction has been reported to increase circulating acylcarnitine levels and suppress CD36 expression in the fetal sheep heart [35]. In parallel, previous studies have shown that harmful external stimuli, such as corn silk, promoted apoptosis by downregulating CD36 expression in HTR-8/SVneo cells, thereby contributing to recurrent miscarriage [36]. These observations further highlighted the importance of CD36 in trophoblast function and pregnancy outcome.

However, several limitations of this study must be acknowledged. Firstly, while our findings demonstrated that *Pg*-OMV exerted dual inhibitory effects on FAO and mitochondrial function in HTR-8/SVneo cells, the mechanistic link between these alterations and trophoblast injury warrants further investigation. In particular, rescue experiments aimed at restoring FAO activity was not performed in the present study; therefore, whether FAO impairment is required for *Pg*-OMV-induced mitochondrial dysfunction remains unresolved. Secondly, although CD36 downregulation was consistently observed, its mechanistic role was not directly examined. Future gain- and loss-of-function studies are necessary to determine whether CD36 functions as an upstream regulator of the metabolic and mitochondrial alterations induced by *Pg*-OMV. Thirdly, the biological significance of Carnitine C20 accumulation remains to be clarified. Targeted metabolomics and metabolite-focused functional studies are required to ascertain whether this change merely serves as a biomarker of impaired FAO or actively contributes to placental injury.

## 5. Conclusions

In conclusion, this study demonstrated that *Pg*-OMV invaded placental trophoblasts and subsequently suppressed mitochondrial FAO, which was primarily manifested by elevated carnitines and decreased CD36 expression. These findings established a mechanistic link between periodontal pathogen-derived vesicles and placental insufficiency, highlighted the inhibition of mitochondrial FAO as a critical contributor to *Pg*-OMV-mediated APOs. This work provided novel insights into the metabolic origins of pregnancy complications associated with periodontitis and underscored the importance of periodontal health during gestation, with implications for future translational and interventional studies in reproductive medicine.

## Data Availability

Data will be made available on request.

## References

[1] Curtis M.A., Diaz P.I., Van Dyke T.E. (2020). The role of the microbiota in periodontal disease. Periodontology 2000.

[2] Sanz M., Kornman K. (2013). Periodontitis and adverse pregnancy outcomes: Consensus report of the Joint EFP/AAP Workshop on Periodontitis and Systemic Diseases. J. Periodontol..

[3] Chen P., Hong F., Yu X. (2022). Prevalence of periodontal disease in pregnancy: A systematic review and meta-analysis. J. Dent..

[4] Boggess K.A., Beck J.D., Murtha A.P., Moss K., Offenbacher S. (2006). Maternal periodontal disease in early pregnancy and risk for a small-for-gestational-age infant. Am. J. Obstet. Gynecol..

[5] Lohana M.H., Suragimath G., Patange R.P., Varma S., Zope S.A. (2017). A Prospective Cohort Study to Assess and Correlate the Maternal Periodontal Status with Their Pregnancy Outcome. J. Obstet. Gynaecol. India.

[6] Wu Z., Long W., Yin Y., Tan B., Liu C., Li H., Ge S. (2025). Outer membrane vesicles of Porphyromonas gingivalis: Recent advances in pathogenicity and associated mechanisms. Front. Microbiol..

[7] León R., Silva N., Ovalle A., Chaparro A., Ahumada A., Gajardo M., Martinez M., Gamonal J. (2007). Detection of Porphyromonas gingivalis in the amniotic fluid in pregnant women with a diagnosis of threatened premature labor. J. Periodontol..

[8] Hirohata N., Komine-Aizawa S., Tamura M., Ochiai K., Sugitani M., Hayakawa S. (2017). Porphyromonas gingivalis Suppresses Trophoblast Invasion by Soluble Factors. J. Periodontol..

[9] Katz J., Chegini N., Shiverick K.T., Lamont R.J. (2009). Localization of P. gingivalis in preterm delivery placenta. J. Dent. Res..

[10] Barak S., Oettinger-Barak O., Machtei E.E., Sprecher H., Ohel G. (2007). Evidence of periopathogenic microorganisms in placentas of women with preeclampsia. J. Periodontol..

[11] Chopra A., Radhakrishnan R., Sharma M. (2020). Porphyromonas gingivalis and adverse pregnancy outcomes: A review on its intricate pathogenic mechanisms. Crit. Rev. Microbiol..

[12] Gong T., Chen Q., Mao H., Zhang Y., Ren H., Xu M., Chen H., Yang D. (2022). Outer membrane vesicles of Porphyromonas gingivalis trigger NLRP3 inflammasome and induce neuroinflammation, tau phosphorylation, and memory dysfunction in mice. Front. Cell. Infect. Microbiol..

[13] Lara B., Loureiro I., Gliosca L., Castagnola L., Merech F., Gallino L., Calo G., Sassot M., Ramhorst R., Vota D. (2023). Porphyromonas gingivalis outer membrane vesicles shape trophoblast cell metabolism impairing functions associated to adverse pregnancy outcome. J. Cell. Physiol..

[14] Chuang W.C., Yang C.N., Wang H.W., Lin S.K., Yu C.C., Syu J.H., Chiang C.P., Shiao Y.J., Chen Y.W. (2024). The mechanisms of Porphyromonas gingivalis-derived outer membrane vesicles-induced neurotoxicity and microglia activation. J. Dent. Sci..

[15] Seyama M., Yoshida K., Yoshida K., Fujiwara N., Ono K., Eguchi T., Kawai H., Guo J., Weng Y., Haoze Y. (2020). Outer membrane vesicles of Porphyromonas gingivalis attenuate insulin sensitivity by delivering gingipains to the liver. Biochim. Biophys. Acta Mol. Basis Dis..

[16] Maltepe E., Fisher S.J. (2015). Placenta: The forgotten organ. Annu. Rev. Cell Dev. Biol..

[17] Zhang L., Wang Z., Wu H., Gao Y., Zheng J., Zhang J. (2022). Maternal High-Fat Diet Impairs Placental Fatty Acid β-Oxidation and Metabolic Homeostasis in the Offspring. Front. Nutr..

[18] Rector R.S., Ibdah J.A. (2010). Fatty acid oxidation disorders: Maternal health and neonatal outcomes. Semin. Fetal Neonatal Med..

[19] Fan R., Zhou Y., Chen X., Zhong X., He F., Peng W., Li L., Wang X., Xu Y. (2023). Porphyromonas gingivalis Outer Membrane Vesicles Promote Apoptosis via msRNA-Regulated DNA Methylation in Periodontitis. Microbiol. Spectr..

[20] Lara B., Sassot M., Calo G., Paparini D., Gliosca L., Chaufan G., Loureiro I., Vota D., Ramhorst R., Pérez Leirós C. (2023). Extracellular Vesicles of Porphyromonas gingivalis Disrupt Trophoblast Cell Interaction with Vascular and Immune Cells in an In Vitro Model of Early Placentation. Life.

[21] Kim J.Y., Suh J.W., Kang J.S., Kim S.B., Yoon Y.K., Sohn J.W. (2023). Gram-Negative Bacteria’s Outer Membrane Vesicles. Infect. Chemother..

[22] Li S., Li L., Zhang C., Fu H., Yu S., Zhou M., Guo J., Fang Z., Li A., Zhao M. (2023). PM2.5 leads to adverse pregnancy outcomes by inducing trophoblast oxidative stress and mitochondrial apoptosis via KLF9/CYP1A1 transcriptional axis. eLife.

[23] Tao S., Yang M., Pan B., Wang Y., Tian F., Han D., Shao W., Yang W., Xie Y., Fang X. (2023). Maternal exposure to ambient PM(2.5) perturbs the metabolic homeostasis of maternal serum and placenta in mice. Environ. Res..

[24] Otsubo C., Bharathi S., Uppala R., Ilkayeva O.R., Wang D., McHugh K., Zou Y., Wang J., Alcorn J.F., Zuo Y.Y. (2015). Long-chain Acylcarnitines Reduce Lung Function by Inhibiting Pulmonary Surfactant. J. Biol. Chem..

[25] Yang X., Okamura D.M., Lu X., Chen Y., Moorhead J., Varghese Z., Ruan X.Z. (2017). CD36 in chronic kidney disease: Novel insights and therapeutic opportunities. Nat. Rev. Nephrol..

[26] Seok J., Jung H.S., Park S., Lee J.O., Kim C.J., Kim G.J. (2020). Alteration of fatty acid oxidation by increased CPT1A on replicative senescence of placenta-derived mesenchymal stem cells. Stem Cell Res. Ther..

[27] Merech F., Lara B., Rios D., Paparini D., Ramhorst R., Hauk V., Pérez Leirós C., Vota D. (2025). Vasoactive intestinal peptide induces metabolic rewiring of human-derived cytotrophoblast cells to promote cell migration. Biochim. Biophys. Acta Mol. Cell Res..

[28] Adeva-Andany M.M., Calvo-Castro I., Fernández-Fernández C., Donapetry-García C., Pedre-Piñeiro A.M. (2017). Significance of l-carnitine for human health. IUBMB Life.

[29] Dambrova M., Makrecka-Kuka M., Kuka J., Vilskersts R., Nordberg D., Attwood M.M., Smesny S., Sen Z.D., Guo A.C., Oler E. (2022). Acylcarnitines: Nomenclature, Biomarkers, Therapeutic Potential, Drug Targets, and Clinical Trials. Pharmacol. Rev..

[30] Visiedo F., Vázquez-Fonseca L., Ábalos-Martínez J., Broullón-Molanes J.R., Quintero-Prado R., Mateos R.M., Bugatto F. (2023). Maternal elevated inflammation impairs placental fatty acids β-oxidation in women with gestational diabetes mellitus. Front. Endocrinol..

[31] Cerullo D., Mantzouratou P., Lavecchia A.M., Balsamo M., Corna D., Brunelli L., Xinaris C. (2025). Triiodothyronine protects infarcted myocardium by reducing apoptosis and preserving mitochondria. Basic Res. Cardiol..

[32] Zhu Y., Ma Y., Li W., Pan Y., Wang Y., Ye Q., Pan Y., Xiang Y., Jiang P., Fang Y. (2025). Targeting GPX2 to disrupt lipid homeostasis and enhance cisplatin sensitivity in diffuse gastric cancer. Cell Death Discov..

[33] Huang S., Cao G., Dai D., Xu Q., Ruiz S., Shindo S., Nakamura S., Kawai T., Lin J., Han X. (2023). Porphyromonas gingivalis outer membrane vesicles exacerbate retinal microvascular endothelial cell dysfunction in diabetic retinopathy. Front. Microbiol..

[34] Liu S., Zhang H., Li Y., Zhang Y., Bian Y., Zeng Y., Yao X., Wan J., Chen X., Li J. (2021). S100A4 enhances protumor macrophage polarization by control of PPAR-γ-dependent induction of fatty acid oxidation. J. Immunother. Cancer.

[35] Drake R.R., Louey S., Thornburg K.L. (2022). Intrauterine growth restriction elevates circulating acylcarnitines and suppresses fatty acid metabolism genes in the fetal sheep heart. J. Physiol..

[36] Zheng Z., Lin X., Ma F., Shi Q., Li X., Wang Y., Li H., Ge R.S., Pan P. (2025). Zearalenone impairs trophoblast function via ROS-mediated apoptosis and autophagy: A mechanistic insight into unexplained recurrent spontaneous abortion. Ecotoxicol. Environ. Saf..

